# Agronomic Practices and Performances of Quinoa under Field Conditions: A Systematic Review

**DOI:** 10.3390/plants10010072

**Published:** 2020-12-31

**Authors:** Mohamed Houssemeddine Sellami, Cataldo Pulvento, Antonella Lavini

**Affiliations:** 1 Institute for Agricultural and Forestry Systems in the Mediterranean (ISAFOM), National Research Council of Italy (CNR), 80055 Portici (NA), Italy; mohamed.sellami@isafom.cnr.it (M.H.S.); antonella.lavini@isafom.cnr.it (A.L.); 2 Institute for Biosciences and Bioresources (IBBR), National Research Council of Italy (CNR), Via Amendola, 165/A, 70126 Bari, Italy

**Keywords:** quinoa, systematic review, bibliometric analysis, concept network analysis, agronomic practices

## Abstract

Quinoa (*Chenopodium quinoa* Willd.) is one of the most popular emerging food crops in the Andean region. It is tolerant to environmental stresses and characterized by interesting nutritional traits. Thus, it has the potential to contribute to food and nutrition security in marginal environments. In this study, we conducted a systematic review integrated with a bibliometric analysis of cropping practices of quinoa under field conditions. The analysis is based on published data from the literature relating to the period 2000–2020. A total of 33 publications were identified, revealing that scientific research on the agronomic practices and performances of quinoa under field conditions is still limited. Africa, Asia, and Europe were the leading research production sites in this field and together provided over 81% of the total scientific production. There were no papers from the Australian continent. The number of papers screened dealing with tillage and weed control management was very limited. The keyword co-occurrence network analyses revealed that the main topics addressed in the scientific literature related to the effect of “variety” and “deficit irrigation”, followed by “water quality”, “fertilization”, and “sowing date” on seed yield. Results from this study will permit us to identify knowledge gaps and limited collaboration among authors and institutions from different countries. Salinity, sowing density, and sowing date were the agronomic interventions affecting productive response the most.

## 1. Introduction

In recent years, quinoa (*Chenopodium quinoa* Willd.) cultivation has expanded to several countries beyond its area of origin due to increasing interest, market development, research, and promotion [1]. Thanks to its high-quality protein content, quinoa is considered a promising candidate for enhancing high-quality plant protein food production in the world [2]. It is recognized as a crop of great value in terms of tolerance to abiotic stresses and it is one of the most nutritious food crops currently known [3]. However, there are still many issues, including a lack of knowledge of best management practices, which need to be addressed in order to introduce quinoa crop in marginal areas [4].

Till the early 1900s, the cultivation of quinoa remained limited to its countries of origin. In the following century, quinoa arrived in Africa, Asia, Europe, and North America [1]. Until 1999, few studies were published in peer-review journals concerning agronomic practices in the open field. Risi and Galewey [5] evaluated the effect of sowing density and sowing date on different genotypes of quinoa grown in the UK. Jacobsen et al. [6] analyzed the effects of varying the nitrogen fertilization rate, seed rate, row spacing, harvesting method, and harvest date on quinoa in Denmark. In 1998, Vacher [7] analyzed the effect of drought on quinoa in Bolivia. Besides these few examples, most of the studies carried out before the new millennium on quinoa were reported in the so-called gray literature; many of the experimental trials were conducted in the countries of origin, but not available in international databases.

Despite the importance of quinoa in marginal areas, its adaptability to unfavorable environments, high protein content, and the interesting nutritional properties of the seed, few studies have been conducted on its yield responses to different strategies of agronomic management under field conditions; this represents a gap in research in this field.

Therefore, there is a need for a systematic review integrated with a bibliometric analysis to answer the question: what is the research gap in agronomic management and performance of quinoa under field conditions?

A systematic review (SR) is defined as a review of the evidence on a formulated question that uses systematic and explicit methods to identify, select, and critically appraise relevant primary research; it is used to extract and analyze data from the studies included in the review [8]. Bibliometric or research impact is the quantitative method of analyzing citations and content for academic journals, books, and researchers. The quantitative impact of a given publication is appraised by measuring the number of times a certain work is cited by other resources [9].

This study aims to apply a systematic review integrated with a bibliometric analysis to evaluate the research trend of the last twenty years (2000–2020) on the subject of cropping practices of quinoa production under field conditions.

## 2. Results

### 2.1. Screening Process

**Figure 1:**
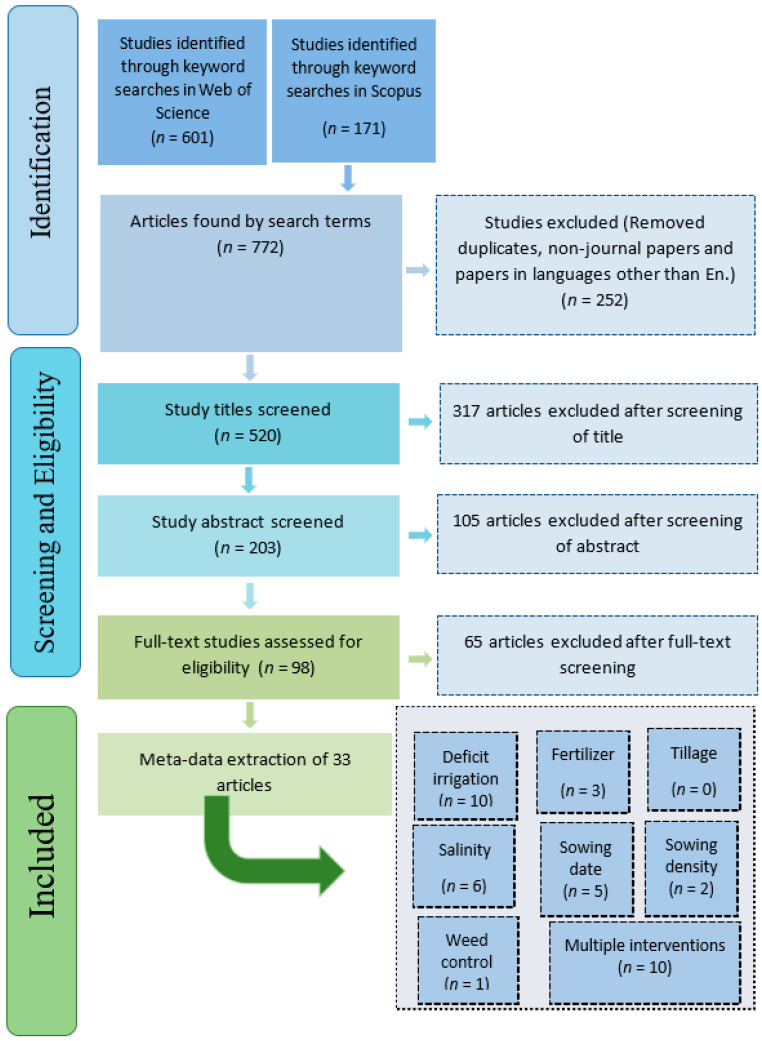
Selection of studies for inclusion in the systematic review (*n*—the number of studies).

In total 520 sources of literature were identified within academic databases (after the removal of 252 duplicates or non-journal papers), of which 33 were selected and analyzed (Table 1), to provide 513 observations. The screening process is described in Figure 1.

### 2.2. Evolution Articles over the Years

**Figure 2:**
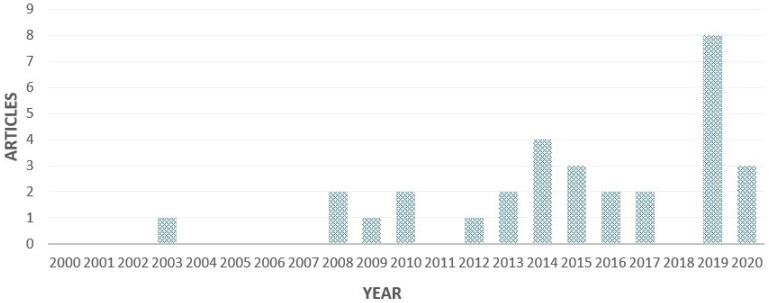
Annual scientific production.

Figure 2 shows the annual scientific production dealing with the agronomic practices and performances of quinoa under field conditions in the world. The research on the effect of agronomic practices on quinoa under field conditions is considered relatively young and started less than twenty years ago. In fact, the first paper in this research area was published in 2003. The number of published research papers has fluctuated over the last two decades, reaching a peak of eight during 2019. This leads us to infer that this rise in the number of articles over the years represents an increasing interest in this research area.

### 2.3. Geographical Distribution of Articles

**Figure 3:**
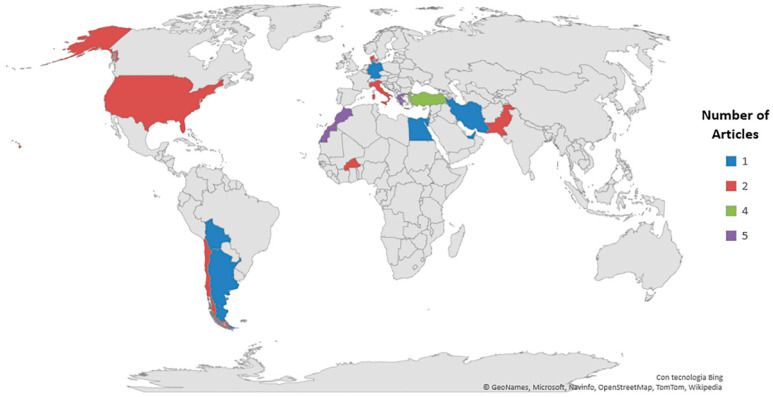
Number of published research articles worldwide.

Our screening process reveals that accessible published research on the agronomic practices and performances of quinoa under field conditions in the world, with high reporting standard suitable for this systematic review, is concentrated in Europe (13 articles, 39%), followed by Africa (8 articles, 24%), and Asia (6 articles, 18%) (Table 2, Figure 3). These three continents together represent more than 80% of the research papers published in the past two decades. Such research is lacking in the Australian continent, a large part of which is arid.

In the following text, numbers in brackets indicate the number of research articles published in the categories described. Greece and Morocco (*n* = 5) were the most frequently studied countries, followed by Turkey (*n* = 4) (Figure 3).

The most commonly studied climatic zones were Csa (*n* = 13) with a hot-summer Mediterranean climate (Table 3).

### 2.4. Management and Duration of Trials

**Figure 4:**
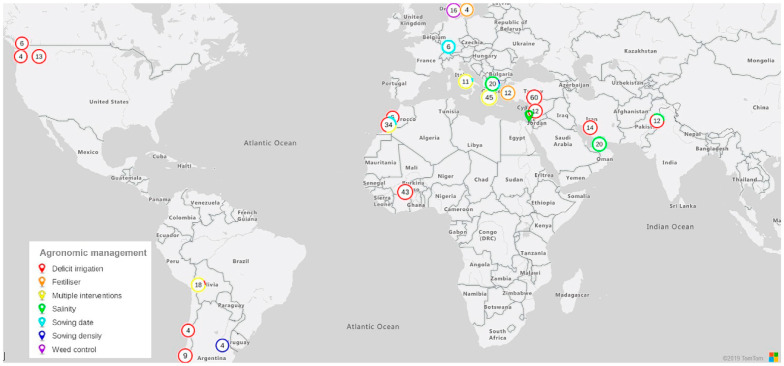
World map showing the number of observations per country. The writing and color in each Doughnut chart represent the total number of observations for each study area and interventions (deficit irrigation, salinity, fertilizer, sowing date, sowing density, weed control, and multiple interventions), respectively.

Seven main groups of agronomic management were identified during the screening: deficit irrigation (*n* = 10), salinity (*n* = 6), fertilization (*n* = 3), sowing density (*n* = 2), sowing date (*n* = 5), weed control (*n* = 1), and multiple interventions (*n* = 10). The number of articles and observations reporting investigations of each group of treatments is shown in Figure 3 and Figure 4, and Table 3.

**Figure 5:**
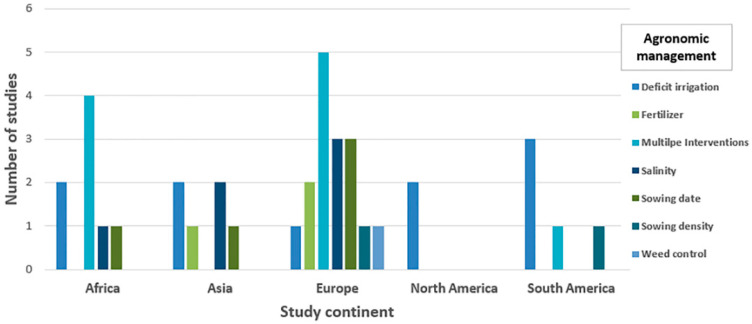
The number of studies undertaken across continents. The numbers are separated by the agronomic management group investigated in each study. Studies may be present in more than one agronomic management category.

In the continent of Europe we found all types of agronomic management, with salinity, sowing date, and multiple interventions (e.g., tillage and fertilization) being the most frequently studied treatments (3, 3, and 5 articles, respectively), while in North America, only deficit irrigation was found (2 articles) (Figure 5).

A major proportion of the studies were carried out in temperate zones (59% of the total observations), of which 35% were deficit irrigation and 37% multiple interventions (e.g., tillage/fertilization; deficit irrigation/salinity). Salinity treatment represented 50% of the total observations in the arid zones, while 100% of the total observations in the tropical zones were represented by multiple interventions (e.g., deficit irrigation and fertilization) (Table 4).

**Figure 6:**
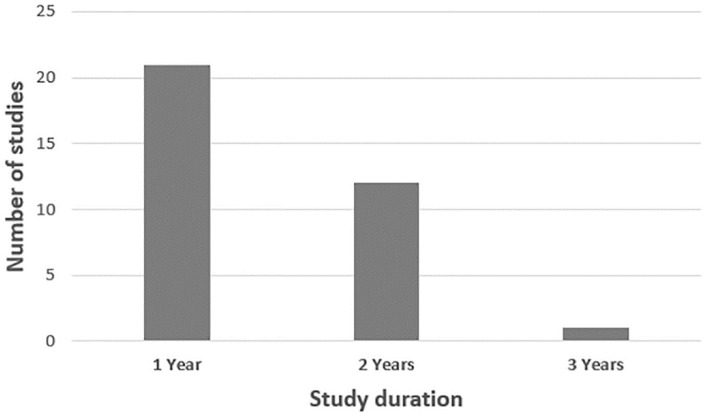
Duration of trials included in the systematic review.

A large number of studies were carried out over one year (21) and less commonly over two years (12), with only one study carried out over three years (Figure 6). The longest studies occurred in Europe (three years) with tillage and fertilization treatments in the Agrinio area, Greece (Table 1).

### 2.5. Most Productive Institutions and Analysis of Source Publications

**Figure 7:**
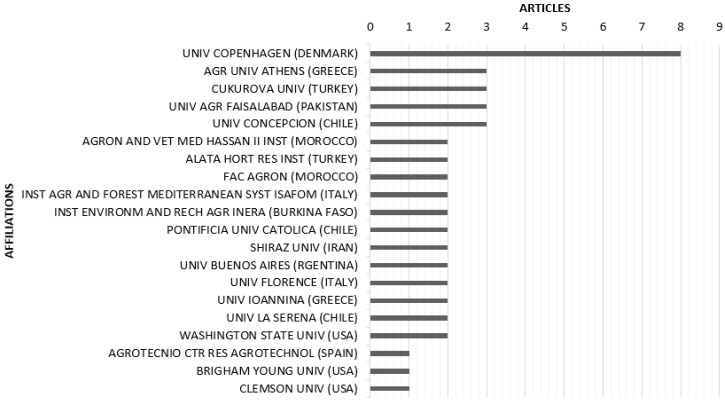
The top 20 most productive institutions in terms of publications.

Figure 7 shows the top twenty most productive institutions. According to the bibliometric analysis, the most productive institutions were the University of Copenhagen, Denmark with eight articles, followed by the Agricultural University of Athens, Greece; Çukurova University, Turkey; University of Agriculture Faisalabad, Pakistan; and the University of Concepción, Chile, who produced three articles each.

**Figure 8:**
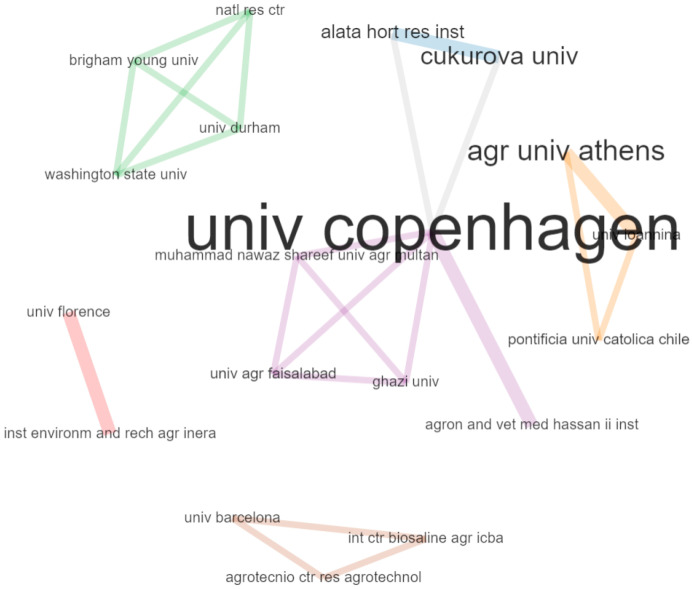
Institution collaboration network. The total number of papers related to each institution proportionally sizing the corresponding label. The thicker the line, the closer the connection between the two institutions.

Figure 8 shows the collaboration network among the leading institutions. The network was drawn from the institution × institution adjacency matrix counting the co-authored publications. In the open-source R package bibliometrix [43], we considered only the first 20 institutions, with a threshold of at least one co-authored publications. The institutions were classified into six clusters, with the first cluster formed by the University of Florence, Italy, and Institut de l’Environnement et de Recherches Agricoles, Burkina Fasso, who are closely connected to each other. A similar situation was observed in respect of two institutions from Turkey, the Alata Horticultural Research Institute and Çukurova University. The third cluster represented a strong connection between two US universities, Washington State University and Brigham Young University, with the National Research Centre in Egypt as well as Durham University in the UK. Another cluster was represented by the University of Copenhagen, Denmark, which is closely connected to the Hassan II Institute of Agronomy and Veterinary Medicine, Morocco, and weakly connected to the Pakistan universities. The fifth cluster is represented by the different Greek universities connected to the Pontifical Catholic University of Chile. Finally, the Agrotecnio Centre for Research in Agrotechnology and the University of Barcelona in Spain were connected with the International Center for Biosaline Agriculture in the UAE.

This analysis is useful for identifying potential partners and cooperative organizations and opening up prospects for research cooperation in this field.

**Figure 9:**
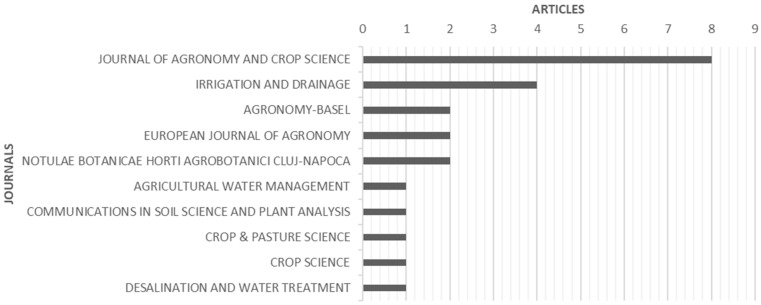
Top 10 journals that published articles related to the agronomic practices and performances of quinoa under field conditions.

The bibliometric analysis showed that between 2000 and 2020, the 33 papers included in the systematic review were published in 20 journals. Figure 9 shows the top 10 journals that published articles related to the agronomic practices and performances of quinoa under field conditions. According to the analysis, the journals mostly selected by authors were the Journal of Agronomy and Crop Science followed by Irrigation and Drainage.

### 2.6. Concept Network Analysis

#### 2.6.1. Terms Analysis

**Figure 10:**
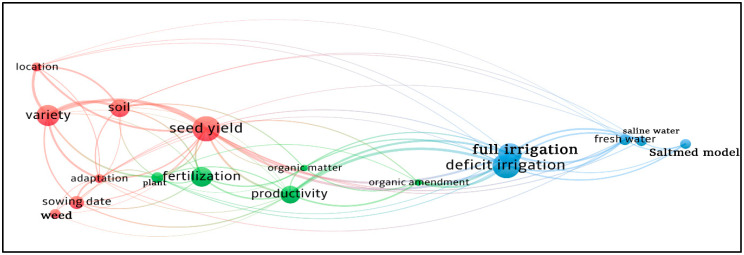
Concept network map for the titles of the 33 articles included in the systematic review and the corresponding abstracts. Map produced by VOSviewer.

A concept network analysis was performed to extract the terms most used in the title and abstract fields of the publications selected for final review in this research. Our bibliometric analysis revealed the presence of 1206 terms used in the 33 articles. The minimum number of occurrences of a term used in VOSviewer software [44] was set to 6. Accordingly, of the 1206 terms, 17 met the threshold. Figure 10 shows the concept network map for the titles of the 33 articles included in the systematic review and the corresponding abstracts. The 17 terms are classified into three different clusters, in which these terms are based on the same topics (co-occurrence). The higher the number of co-occurrences of two terms, the closer will they be located on the map. The mapped data revealed that the quinoa research fields are related to the effect of the agronomic management (water quality, deficit irrigation, fertilization, sowing date), variety, and soil on seed yield of quinoa. The terms with the highest occurrences are seed yield, variety, deficit irrigation, full irrigation, and fertilization, with a number of occurrences of 23, 19, 25, 20, and 18, respectively.

#### 2.6.2. Authors Keywords Analysis

**Figure 11:**
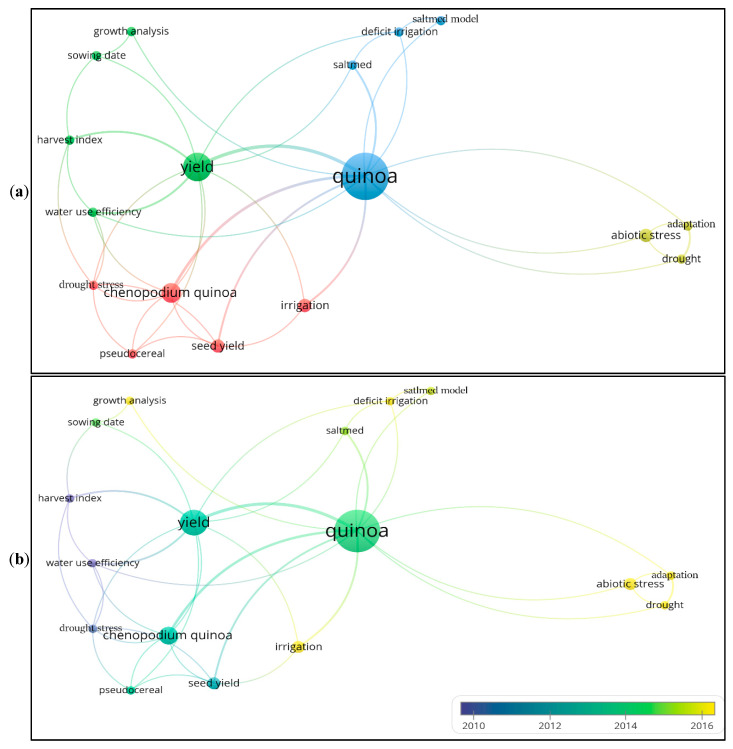
Authors keywords maps. (**a**) Network visualization of authors keywords co-occurrence. (**b**) Thematic evolution of authors keywords in the field of research on the agronomic practices and performances of quinoa under field conditions 2000–2020. Map produced by VOSviewer.

Figure 11a shows the author’s keywords map. This analysis was performed by VOSviewer. The minimum number of occurrences of a keyword was set to two. Consequently, of the 110 identified keywords, 17 met the threshold. This analysis identified four clusters: quinoa (modeling), seed yield, crop (*Chenopodium quinoa*) and abiotic stress (Figure 11a).

Figure 11b shows the thematic evolution of the author’s keywords based on the average times they appeared in our collection of articles. We find that the keywords related to water use efficiency, harvest index, and drought stress appeared early while those related to deficit irrigation, abiotic stress, and growth analysis appeared later.

### 2.7. Overall Yield Across Factors of Variation

**Figure 12:**
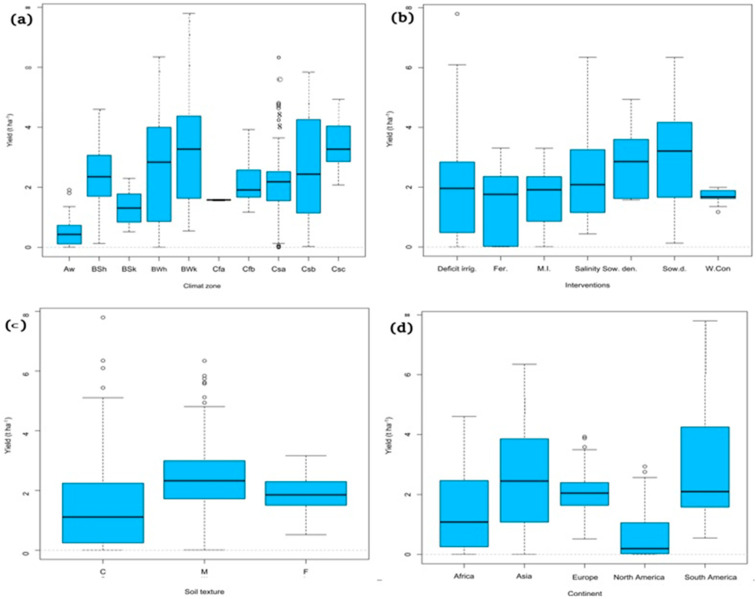
Box plots of patterns of yield (t ha^−1^) for all articles (*n* = 33) across: (**a**) different groups of climatic zones, Aw: tropical wet-dry climate; BSh: hot semi-arid climate; BSk: cold semi-arid climate; BWh: hot desert climate; BWk: cold desert climate; Cfa: humid subtropical; Cfb: maritime; Csa: interior Mediterranean; Csb: coastal Mediterranean; and Csc: cold-summer Mediterranean climate, (**b**) different groups of agronomic management, A: deficit irrigation; AB: deficit irrigation and salinity; AD: deficit irrigation and fertilizer; B: salinity; C: tillage; CD: tillage and fertilizer; D: fertilizer; E: sowing density; EF: sowing density and sowing date; F: sowing date; and G: weed control, (**c**) different groups of soil texture, C: coarse soil; M: medium soil; F: fine soil, and (**d**) different groups of the continent. Box edges represent the upper and lower quantile with the median value shown in the middle of the box. The small circles on the box plot relate to outliers.

Figure 12 shows that the variation of yield due to environment, agronomic management, and soil factors was quite large. Yield response varied across the ten climatic zones with the lowest value of 0 t ha^−1^ obtained in the tropical climates zone (Aw) and the highest value of 7.80 t ha^−1^ observed in the cold desert climate zone (BWk). It can be seen from Figure 12a that the highest seeds yield was recorded in the arid climate zone and the lowest in the tropical climate zone. The humid subtropical zone (Cfa) was the most homogeneous class, followed by the Aw zone. The hot desert climate zone (BWh) and the coastal Mediterranean zone (Csb) were the least homogeneous classes. The yield values for zones Aw, BSk, and Cfa were clearly behind those of BWh, BWk, and Csb.

Table 4 and Figure 12b show the yield variation between different agronomic managements. The highest value of 6.35 t ha^−1^ was obtained in the salinity and sowing date treatment. Salinity, sowing density, and sowing date treatments were the agronomic interventions most influential to productive response; fertilization and multiple interventions were less impactful, with average yield values ranging from 1.40 and 1.67 t ha^−1^, respectively.

The variation of yield between soil texture showed that medium soil (M) and fine soil (F) were the most productive, with average yield values ranging from 2.36 to 1.83 t ha^−1^, respectively. The highest value of 7.80 t ha^−1^ was obtained in the coarse soil (C), with fine soil (F) being the most homogeneous class (Figure 12c).

Studies conducted in South America and Asia showed the highest yields, ranging from 0.55 to 7.80 t ha^−1^, and 0.01 to 6.35 t ha^−1^, respectively (Figure 12d). In contrast, the yield values of the North American continent were clearly behind other continents. The European continent was the most homogeneous class, with yield values ranging from 0.52 to 3.93 t ha^−1^.

## 3. Discussion

The systematic review integrated with bibliometric analysis allows the identification of a research gap in the cropping practices of quinoa production under field conditions, highlighting the necessity to develop research and to establish global research networks in order to include different scientists worldwide, especially from arid regions.

The analysis of co-occurrence terms and the author’s keywords identified the main topics of actual research. Over the last 20 years, of the top 17 terms in the 1206 used in 33 articles, five (seed yield, variety, deficit irrigation, full irrigation, and fertilization) registered the highest co-occurrence frequency, indicating that from 2000 to 2020, research was primarily focused on these topics. The high occurrence of the terms seed yield and deficit irrigation in the titles and abstracts of the analyzed papers may indicate the focus of most of the papers on the effects of deficit irrigation on seed yield. According to Radhakrishnan et al. [45], the keyword co-occurrence network analyses can be performed quickly to explore a wide range of literature and can provide a knowledge map and insights before conducting a rigorous conventional systematic review. In the early stages of research on the cropping practices of quinoa production under field conditions, the studies focused on topics related to water use efficiency, harvest index, and drought stress. Later on, the focus was on more recent topics related to deficit irrigation and abiotic stress. In the last twenty years, drought signals in the field have been confirmed by a large number of field studies [46]. The major agricultural use of water is for irrigation, which is thus affected by decreased supply. In recent years, significant effort was made to increase the efficiency of water use through the use of deficit irrigation strategies [47].

The bibliographic analysis of the selected studies highlighted the beginning of the study of best agronomic practices for quinoa production in 2003, probably due to the impact of specific research projects conducted from 1990 to 2000 [48]. The number of studies strongly increased after 2013, when the FAO celebrated the International Year of Quinoa; this activity of disseminating the importance of quinoa as a crop resistant to unfavorable environments and a high-quality protein source focused world attention on it [49].

The highlighting of the resistance of quinoa to abiotic stresses and its adaptability to different environments led to an increase in studies, especially in countries characterized by a hot, arid climate and water scarcity (Morocco, Egypt, Burkina Faso, and the UAE) and countries at risk of water and salt stress due to climate change (Italy, Greece, Turkey, Pakistan, and the US). Many of the studies in these countries were related to the evaluation of the effect of deficit irrigation and the use of saline water on quinoa. Much importance has also been bestowed on the study of the best time and sowing density, which represent the main agronomic practices for the introduction of a crop in a new environment.

The analyzed papers showed that quinoa is able to guarantee seed yields in line with those of the countries of origin even in different climatic conditions and different soils texture; in fact, even if quinoa prefers well-drained soils and warm beds [50], it has been shown to adapt well and guarantee high yields even in clayey and less-drained soils.

The geographical distribution of the studies confirmed that quinoa adapts to different pedo-climatic regions.

The papers analyzed have also shown that agronomic practices such as sowing date and sowing density are very important parameters that affect seed yield; the evaluation of the best sowing date is fundamental in the case of quinoa introduction in new environments.

Studies on the effect of irrigation management, such as deficit irrigation or use of saline water, have confirmed that these are sustainable practices conducive to the optimization of production by reducing the use of water resources; moreover, quinoa can be grown in marginal environments [51] characterized by scarcity and poor-quality of water.

The total number of papers found in peer-reviewed journals appears still limited compared to the potential of quinoa to adapt to different environments and the great genetic diversity that distinguishes this crop. Today, it is estimated that there are over 6000 varieties cultivated by farmers [52], each of these accessions with distinctive genetic characteristics requiring specific study.

## 4. Materials and Methods 

### 4.1. Literature Research

A systematic review (SR) was conducted across two bibliographic databases (ISI Web of Science™ and Scopus™), to identify studies related to the agronomic practices of quinoa (*Chenopodium quinoa*) production in the World. The studies were published between 2000 and 2020 in peer-reviewed journals written in English. The searches of academic databases were performed on 5 October 2020. In bibliographic databases the following search strings were used to search “topic words” combined with Boolean operators: ((field OR cultivar* OR genotyp* OR crop* OR farm* OR cultivat* OR accessions) AND (yield OR grain OR product* OR seed*) AND (quinoa or (Chenopodium and quinoa))). The wildcards * represent any number of characters.

### 4.2. Inclusion and Exclusion Criteria

We used a highly robust and rational systematic review methodology to synthesize the evidence from a wide range of sources. In this study, we constrained the SR by defining boundaries to include: (I) studies conducted only under field conditions, but not under glasshouse conditions and pots; and (II) studies that focused on crop productivity, omitting forestry, fisheries, livestock, and other non-food crop agricultural sectors. Following the SR convention, the search terms were based on the four PICO components (population, intervention, comparator, and outcome) (Table 5). A list of references included in the SR meta-database is provided in Table 1.

### 4.3. Screening

Following the removal of duplicates, in order to extract yield information data from accepted papers were entered into Endnote (online bibliographic management software) (version basic; Clarivate Analytics, https://access.clarivate.com/#/login?app=endnote); all the references were retrieved and screened for relevance using the following inclusion criteria: every selected study was screened through three stages: title, abstract, and full text. At each level, records containing or likely to contain relevant information were identified and taken to the next stage.

### 4.4. Coding and Data Extraction

Meta-data (descriptive categorical information regarding citations, study setting, design, and methods) were extracted from included studies following full-text assessment.

The investigated treatments (agronomic management) were recorded for each study as categorical variables where possible; in this case, a complete disjunctive coding of our variables (treatments investigated) was carried out. This means that variables are dichotomous, assuming the value “1” should the keyword be associated with the paper, and “0” if not. This coding was done according to methods described by Cuccurullo et al. [53].

Data for different years or experimental conditions (i.e., cultivars or other experimental factors) within each publication were treated as independent observations. Data were obtained directly from tables and if data were provided in graphical form, means were extracted using WebPlotDigitizer [54].

### 4.5. Bibliometric Analysis and Concept Network Analysis

The meta-data from SR was analyzed for the year of publication, the journal, and the frequency of terms and keywords used by authors. This analysis was carried out with the aid of a software package of comprehensive science mapping analysis, bibliometrix [43] in R studio software [55]. The package is available through the Comprehensive R Archive Network (CRAN, https://cran.r-project.org). We used the VOSviewer software developed by scientometricians [44] (http://www.vosviewer.com) for concept network mapping to generate keywords and term maps.

## 5. Conclusions

This study analyzed the scientific production in the last 20 years on the adoption of agronomic practices for the cultivation of quinoa under field conditions. The results showed that since 2003 there has been a fair amount of scientific production; however, only after 2013, the International Year of Quinoa celebrated by FAO, was there a significant increase in the number of papers.

In many cases, the results of previous experiments mostly carried out in South America have been published in journals and/or volumes not indexed.

The analysis revealed a greater interest in studying the date and density of sowing, and irrigation (deficit irrigation and with saline waters) as sustainable practices to ensure good yields in different environments. The studies analyzed have emphasized that the best agronomic practices can guarantee good production of quinoa even in marginal environments or those characterized by abiotic stress (drought and salinity). Studies have shown that quinoa can also be grown on fine, less-drained soils.

Data reported in the screened paper need to be analyzed more deeply; a useful approach to this could be a meta-analysis using the relative yield as an effect-size estimator. The meta-analysis would allow investigation of the interaction between different continental regions, environmental and agronomic management, and the effect of these factors on yield response.

Scientific production still appears limited and no publications relating to experiments were found for the Australian continent, which is characterized by large arid areas and marginal environments.

Several agronomic practices should be explored such as weed control (only one article has been detected), use of wastewater, soil processing, etc.

This systematic review can be used by researchers to identify deficiencies and best practices in research methodology, fostering collaboration, especially between arid country researchers, increase the field research, and exploit research results at the maximum extent. It would make a significant contribution to the expansion of quinoa in different environments.

## Figures and Tables

**Table 1:** Description of the experimental studies in the selected references.

N°	References	No. of Observations	Location	Climatic Zone	Soil Texture	Year	No. of Genotypes	Yield Range (t ha^−1^)	Agronomic Management
1	Jacobsen et al. [10]	18	Tastrup (DK)	Cfb	Coarse	2004; 2005	1	1.17–1.99	Weed Control
2	Pulvento et al. [11]	12	Vitulazio (IT)	Csa	Medium	2009; 2010	1	1.96–3.1	[Deficit irrigation; Salinity]
3	Jacobsen and Christiansen [12]	6	Tastrup (DK)	Cfb	Coarse	2007–2009	1	1.74–2.27	Fertilization
4	Pulvento et al. [13]	2	Vitulazio (IT)	Csa	Medium	2006	1	1.5–3.28	Sowing date
5	Geren [14]	14	Bornova (TR)	Csa	Medium	2013; 2014	1	0.87–3.31	Fertilization
6	Ince Kaya and Yazar [15]	14	Adana (TR)	Csa	Fine	2010; 2012	1	1.51–2.99	Deficit irrigation; Salinity
7	Yazar et al. [16]	31	Adana (TR)	Csa	Fine	2010–2012	1	1.28–3.17	[Deficit irrigation; Salinity]
8	Alvar-Beltrán et al. [17]	13	Bobo Dioulasso (BF)	Aw	Coarse	2017; 2018	1	0.23–1.36	[Deficit irrigation; Fertilization]
9	Alvar-Beltrán et al. [18]	36	Bobo Dioulasso (BF)	Aw	Coarse	2017	2	0.01–1.91	[Deficit irrigation; Fertilization]; Sowing date
10	Asher et al. [19]	24	Avnei Eitan (IL)	Csa	Medium	2016; 2017	6	1.52–6.34	Sowing date
11	Basra et al. [20]	10	Faisalabad (PK)	BWh	Coarse	2010	2	0.01–0.02	Fertilization
12	Fghire et al. [21]	8	Marrakech (MA)	BSh	Coarse	2011; 2012	1	1.10–4.09	Deficit irrigation
13	Hirich et al. [22]	10	Agadir (MA)	BSh	Medium	2012	1	0.13–3.07	Sowing date
14	Hirich et al. [23]	6	Agadir (MA)	BSh	Medium	2011	1	1.70–3.30	[Deficit irrigation; Fertilization]
15	Filali et al. [24]	20	Agadir (MA)	BSh	Medium	2012	5	1.60–4.60	Deficit irrigation
16	Bertero and Ruiz [25]	8	Pergamino (AR)	Csc	Medium	2003	4	2.08–4.94	Sowing density
17	Bilalis et al. [26]	12	Agrinio (GR)	Csa	Medium	2010; 2011	1	2.18–2.64	[Tillage; Fertilization]
18	Çolak et al. [27]	22	Adana (TR)	Csa	Fine	2016; 2017	1	1.86–3.02	Deficit irrigation
19	Eisa et al. [28]	2	Sinai (EG)	BWh	Coarse; Medium	2014	1	0.44–1.15	Salinity
20	Fischer et al. [29]	12	Nuble (CL)	Csb	Medium	2010	3	3.61–5.84	Deficit irrigation
21	Geerts et al. [30]	21	Patacamaya; Condoriri (BO)	BSk	Medium	2005; 2006	1	0.79–2.10	[Deficit irrigation; Fertilization]; [Deficit irrigation.; Sowing date]; Deficit irrigation
22	Hinojosa et al. [31]	12	Pullman (US)	Csa	Coarse	2016	6	0.00–0.96	Deficit irrigation
23	Hinojosa et al. [32]	32	Pullman; Chimacum; Mount Vernon (US)	Csa; Csb	Medium; Coarse	2016; 2017	6	0.01–2.94	Deficit irrigation
24	Hirich et al. [33]	6	Agadir (MA)	BSh	Medium	2011	1	1.70–3.30	[Deficit irrigation; Fertilization]
25	Iqbal et al. [34]	8	Faisalabad; Pindi Bhattian (PK)	BWh	Coarse; Medium	2013	2	1.59–3.06	Salinity
26	Kakabouki et al. [35]	16	Agrinio (GR)	Csa	Medium	2012; 2013	1	1.90–2.63	[Tillage; Fertilization]
27	Kakabouki et al. [36]	24	Agrinio (GR)	Csa	Medium	2011–2013	1	1.90–2.66	[Tillage; Fertilization]
28	Karyotis et al. [37]	16	Larissa (GR)	BSk	Fine	2001	8	0.52–2.30	Salinity
29	Martınez et al. [38]	12	Coquimbo; Ovalle (CH)	BWk	Coarse	2004; 2005	2	0.55–7.80	Deficit irrigation
30	Noulas et al. [39]	24	Larissa (GR)	BSk	Fine	1996; 1998; 2001	10	0.52–2.30	Sowing density; Salinity; Sowing date
31	Präger et al. [40]	8	Stuttgart (DE)	Cfb	Medium	2016	2	2.89–3.93	Sowing date
32	Razzaghi et al. [41]	16	Shiraz (IR)	Csa	Medium	2016; 2017	1	0.26–0.86	Deficit irrigation
33	Rezzouk et al. [42]	38	Dubai (UAE)	BWh	Coarse	2016	19	0.53–6.35	Salinity

Agronomic management in square brackets represents multiple interventions. Köppen–Geiger climate zone: Aw: tropical wet-dry climate; BSh: hot semi-arid climate; BSk: cold semi-arid climate; BWh: hot desert climate; BWk: cold desert climate; Cfa: humid subtropical; Cfb: maritime; Csa: interior Mediterranean; Csb: coastal Mediterranean; and Csc: cold-summer Mediterranean climate. DK: Denmark; IT: Italy; TR: Turkey; BF: Burkina Faso; IL: Israel; PK: Pakistan; MA: Morocco; AR: Argentina; GR: Greece; EG: Egypt; CL: Chile; BO: Bolivia; US: United States of America; CH: Switzerland; DE: Germany; IR: Iran; and UAE: United Arab Emirates. Where two years are separated by a semicolon this indicates that an experience is not repeated each year; where two years are separated by a hyphen this indicates that an experience repeated over time.

**Table 2:** Continent-wise descriptive statistical parameters (minimum, maximum, mean, median, SD, and CV) relative to yield (t ha^−1^) for all agronomic managements.

Continent	No. of Countries	List of Countries	No. of Cases	No. of Observations	No. of Genotypes	Yield (t ha^−1^)					C.V. ^2^ (%)
						Minimum	Maximum	Mean	Median	S.D. ^1^	
Africa	3	Burkina Faso, Egypt, Morocco	8	101	10	0.01	4.60	1.42	1.08	1.27	89
Asia	5	Iran, Israel, Pakistan, Turkey, United Arab Emirates	6	118	29	0.01	6.35	2.55	2.45	1.40	64
Europe	4	Denmark, Germany, Greece, Italy	13	197	14	0.52	3.93	2.02	2.05	0.69	33
North America	1	US	2	44	6	0.002	2.94	0.65	0.20	0.87	134
South America	3	Argentina, Bolivia, Chile	4	53	11	0.55	7.80	2.65	2.10	1.45	58

^1^ Standard deviation; ^2^ Coefficient of variation.

**Table 3:** Number of observations included in the meta-dataset as per the Köppen–Geiger climate zone.

Main Climate Groups	Köppen–Geiger Climate Zone	Name of the Climate Zone	No. of Articles	No. of Observations
Tropical Climates	Aw	Aw: tropical wet-dry climate	2	49
Dry Climates	BSh	BSh: hot semi-arid climate	6	54
	BSk	BSk: cold semi-arid climate	3	58
	BWh	BWh: hot desert climate	4	54
	BWk	BWk: cold desert climate	1	12
Temperate Climates	Cfa	Cfa: humid subtropical	1	3
	Cfb	Cfb: maritime	3	32
	Csa	Csa: interior Mediterranean	13	232
	Csb	Csb: coastal Mediterranean	2	30
	Csc	Csc: cold-summer Mediterranean climate	1	8

**Table 4:** Summary of agronomic managements factors used in the systematic review.

Agronomic Management	Articles	Obs.	Countries	Tropical Obs.	Arid Obs.	Temperate Obs.	Yield (t ha^−1^)
Overall	33	513	16	49	160	304	2.00 ± 1.35
Sowing Date	5	48	5	0	14	34	2.94 ± 1.63
Sowing Density	2	12	2	0	4	8	2.81 ± 1.15
Salinity	6	86	5	0	80	6	2.32 ± 1.43
Deficit Irrigation	10	145	7	0	40	105	1.97 ± 1.57
Fertilization	3	30	3	0	10	20	1.40 ± 1.12
Weed Control	1	18	1	0	0	18	1.69 ± 0.22
Multiple Interventions	10	174	6	49	12	113	1.67 ± 0.89

Obs.: Observations.

**Table 5:** Defining the PICO terms for the research “question” used in this study.

PICO	Description
Population	Agriculture: food crops under field conditions
	World: study included all the countries in all the continents
Intervention	Management included sowing date, sowing density, fertilizer, tillage, salinity, deficit irrigation, and weed control
Comparator	Impacts and/or benefits
Outcomes	Yield, yield gap, potential yield, farmer yield, and attainable yield

## Data Availability

Data sharing not applicable. No new data were created or analyzed in this study. Data sharing is not applicable to this article.
